# Critical State of the Art of Sugarcane Industry Wastewater Treatment Technologies and Perspectives for Sustainability

**DOI:** 10.3390/membranes13080709

**Published:** 2023-07-31

**Authors:** Abdoul Wahab Nouhou Moussa, Boukary Sawadogo, Yacouba Konate, Sayon dit Sadio Sidibe, Marc Heran

**Affiliations:** 1 Laboratoire Eaux Hydro-Systèmes et Agriculture (LEHSA), Institut International d’Ingénierie de l’Eau et de l’Environnement (2iE), Rue de la Science, Ouagadougou 01 BP 594, Burkina Faso; boukary.sawadogo@2ie-edu.org (B.S.); yacouba.konate@2ie-edu.org (Y.K.); 2 Laboratoire Energies Renouvelables et Efficacité Energétique (LaBEREE), Institut International d’Ingénierie de l’Eau et de l’Environnement (2iE), Rue de la Science, Ouagadougou 01 BP 594, Burkina Faso; 3 Institut Européen des Membranes, IEM, UMR-5635, Université de Montpellier, CNRS, Place Eugène Bataillon, CEDEX 5, 34095 Montpellier, France; marc.heran@umontpellier.fr

**Keywords:** perspectives, sugarcane industry, sustainability, technologies, wastewater treatment

## Abstract

The worldwide pressure on water resources is aggravated by rapid industrialization, with the food industry, particularly sugar factories, being the foremost contributor. Sugarcane, a primary source of sugar production, requires vast amounts of water, over half of which is discharged as wastewater, often mixed with several byproducts. The discharge of untreated wastewater can have detrimental effects on the environment, making the treatment and reuse of effluents crucial. However, conventional treatment systems may not be adequate for sugarcane industry effluent treatment due to the high organic load and variable chemical and mineral pollution. It is essential to explore pollution-remediating technologies that can achieve a nexus (water, energy, and food) approach and contribute to sustainable development. Based on the extensive literature, membrane technologies such as the membrane bioreactor have shown promising results in treating sugarcane industry wastewater, producing treated water of higher quality, and the possibility of biogas recovery. The byproducts generated from this treatment can also be recovered and used in agriculture for food security. To date, membrane technologies have demonstrated successful results in treating industrial wastewater. This critical review aims to evaluate the performance of traditional and conventional processes in order to propose sustainable perspectives. It also serves to emphasize the need for further research on operating conditions related to membrane bioreactors for valuing sugarcane effluent, to establish it as a sustainable treatment system.

## 1. Introduction

Sugarcane has been cultivated in India since the fourth century [1]. Nevertheless, New Guinea is the original homeland of sugarcane, which is a crop that necessitates abundant water and sunlight. To irrigate a single hectare of sugarcane annually, more than 14,000 m^3^ of water is needed, which implies an average annual rainfall of 1400 mm/y. With a yearly output of roughly 2 billion tons, sugarcane is among the most extensively cultivated plants worldwide, and around 80% of sugar production is derived from it [2]. With a high employability rate and economic value, the sugar industry raises the tension between sustainability and economy with a strong environmental impact [3,4,5]. Prior to the industrial revolution, the efficient breakdown of cellular components of a sugar plant into raw materials for industry was achieved through artisanal and familial methods [6,7]. The industrial processing of sugarcane began in the early 19th century and has undergone significant evolution due to automation. The byproducts of sugarcane processing, including ethanol and biofuels, have revolutionized the experimentation of new sugarcane crops. This has prompted industrialists to incorporate distilleries into their processing chains, as roughly 90% of sugar industries now produce both sugar and ethanol [8,9]. As a result of this trend, the term “cane industry” is increasingly used instead of “sugar industry”, and “coproducts” is used instead of “byproducts” [10,11]. Molasses and bagasse are considered byproducts in a sugar factory, but the production of ethanol generates additional byproducts such as vinasse and blanquette. Regardless of scale, ethanol production from sugarcane generates nonrecyclable byproducts. The growth of sugar industries globally has significant environmental implications, as large volumes of water are extracted and more than half of it is discharged as wastewater without adequate treatment [12]. One of the primary challenges in wastewater treatment is the handling of effluents from sugarcane industries, given their complexity and high pollution load. These effluents contain physical, chemical, and organic pollutants such as organic matter, suspended solids, and residues of chemicals used during processing or handling. Indeed, chemicals used in the sugarcane industry include NaOH and Na_2_CO_3_, which are used for periodic tank cleaning and are often neutralized with HCl. Additionally, Ca(OH)_2_ and H_3_PO_4_ are used to control the pH and clarify the sugar juice [13]. In order to lower the pH and adjust the color, CO_2_ bubbles and SO_2_ are often injected into the sugar juice. Nonetheless, the utilization of these substances, whether regulated or unregulated, has led to a surge in the levels of chemical and organic contaminants, culminating in the buildup of significant amounts of pollutants in sugar effluents [14]. Various treatment systems are utilized for the treatment of wastewater from sugarcane processing industries. This review aims to compile an inventory of these treatment systems, along with their characteristics and limitations, and to identify suitable treatment technologies for the future. The unutilized byproducts in sugarcane industries such as vinasse and bagasse remain highly loaded with organic pollutants, which can still be valorized through biogas production. In fact, chemical processes seem to be unsuitable for the removal of the high (soluble) organic load in sugarcane industry wastewater compared to biological processes. On the other hand, anaerobic membrane bioreactors, in addition to the possibility of biogas recovery, offer several advantages such as treating highly loaded or highly variable effluents compared to conventional technologies while ensuring the water quality. The focus is on the operational conditions of membrane bioreactors, with the aim of guiding future research towards the implementation of this system for better purification performance on sugarcane industry effluents.

To propose efficient treatment systems, it is essential to understand the characteristics of sugar effluents.

## 2. Characteristics of Sugarcane Industry Wastewater

The sugarcane transformation process is highly complex, generating significant amounts of wastewater comprising liquid and solid discharges from the processing, handling, and transformation of sugarcane. These discharges result from cooling, heating, extraction, and reaction processes, as well as from washing byproducts and the control of other rejected specification byproducts. The quantities and qualities of these discharges are highly variable. As the water passes through chambers and tanks from extraction to sugar crystallization, its pollution load in terms of organic matter and various pollutants significantly increases [15]. Approximately 75% of the total volume of wastewater discharged by sugarcane industries is due to the washing of sugarcane, which also includes washing water from tanks that contain processing residues [13]. On the other side, the defibration and grinding processes, which aim to extract the juice, result in solid waste: bagasse, which is composed of fiber.

Approximately 30% of the sugarcane’s weight is comprised of bagasse, which is mainly utilized as a source of energy via combustion. Other issues can be discussed to isolate active substances or high added value [16], such as sugars, furans and organic acids, but they are still dissolved (and diluted) in the liquid phase. Moreover, the energy self-sufficiency of sugar factories is a major challenge for this industry, and the substitution of primary energy of fossil origin by renewable energy from biomass (bagasse) is the simplest way while still valuing the bagasse ashes as fertilizer. Clarification of the juice through decantation yields additional residues that can be repurposed as fertilizer in agriculture. Through the employment of centrifugation, the sugarcane juice is subjected to crystallization, resulting in molasses as a byproduct, which constitutes approximately 3% of the cane volume. Molasses serves as a precursor for ethanol production, which is achieved via fermentation and dehydration processes.

**Figure 1:**
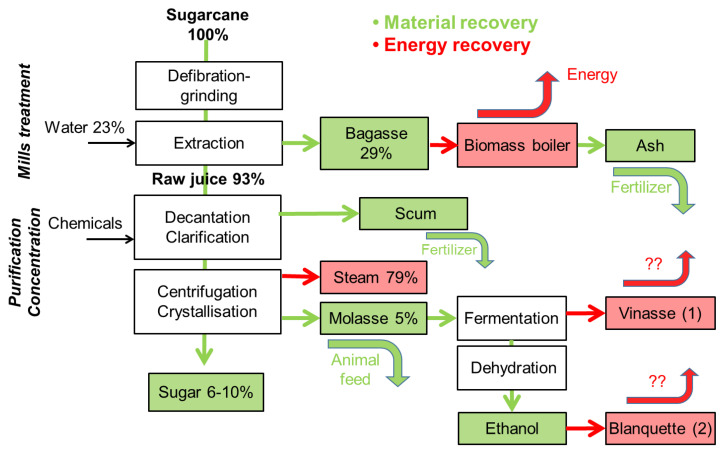
Simplified diagram of a sugarcane.

The production of ethanol from molasses also results in the generation of another byproduct known as vinasse. Additionally, the cleaning of ethanol results in the production of blanquette, which represents another byproduct of the sugarcane industry. It should be noted, however, that blanquette is frequently mixed with vinasse (Figure 1).

The increasing use of ethanol and biofuels derived from sugarcane has diversified energy sources and reduced dependence on fossil fuels, thereby mitigating energy security issues [17,18]. These renewable biofuels offer environmental advantages, including lower carbon footprint, which, by partially replacing traditional fuels, helps to reduce greenhouse gas emissions and combat climate change [19]. Furthermore, the production of ethanol and biofuels from sugarcane creates new economic opportunities, generating additional income and promoting economic development in sugarcane-producing regions [20,21]. The growing demand for these fuels has also stimulated research and development of new technologies to improve production efficiency, biofuel quality, and at least the need for research of the new coproducts (blanquette and vinasse) valorization.

### 2.1. Organoleptic Parameters

The effluent of sugarcane industries undergoes a natural anaerobic decomposition process that often produces unpleasant gases such as CH_4_, CO_2_, NH_3_, and H_2_S, resulting in strong odors and GHG emission. Hydrogen sulfide, in particular, is well known for its foul smell, similar to that of rotten eggs. It is a colorless, inflammable, and toxic gas that can cause health problems when inhaled for extended periods. This odor is often the primary reason for rejecting the wastewater for reuse [22,23]. In wastewater, these odors can be intensified by other volatile compounds, such as indole, skatole, or mercaptans. Poddar et al. [24] provided support for the fact that hydrogen sulfide is soluble in water. However, the solubility of hydrogen sulfide in water is only partial, as stated by [25]. Seasonal temperature variation can indeed affect the level of gas and odor emissions in wastewater. This is because higher temperatures increase the rate of decomposition of organic matter, leading to a greater production of gases such as hydrogen sulfide. In addition, warmer temperatures can also accelerate the growth of odor-producing bacteria, further contributing to the unpleasant odors. Therefore, controlling the temperature of the wastewater treatment process is an important factor in minimizing the release of gases and odors [26]. According to studies conducted by Kaur et al. and Khair et al. [27,28], the odors present in wastewater from sugarcane industries are deemed to be unacceptable.

The presence of ferrous sulfide in wastewater, similar to hydrogen sulfide, can also contribute to the black color of the effluent. If iron is not present, the color may have a different shade [24]. The color of wastewater can be used as an indicator of its age or level of decomposition. Wastewater that is less than 6 h old typically has a light brown color, but as it decomposes, the color tends to shift towards light or medium grey. Several factors can contribute to the brown color of effluents from sugarcane industries, including the caramelization of sugar at high temperatures, acid hydrolysis, phenolics, and melanoidins [29]. Under anaerobic conditions, major bacterial decomposition occurs, leading to the formation of black or dark grey colored wastewater [30]. The coloration of wastewater from sugarcane industries may also result from the presence of humic or fulvic acids, as well as chemical dyes used in the sugarcane transformation processes, which will impart the color of the dye to the wastewater. The impact of wastewater coloration on the environment is significant, as it can directly inhibit photosynthesis and have negative consequences for aquatic life. Sahu et al. [31] reported that the color of sugarcane industry effluent is dark yellow. Li et al. [32] successfully removed approximately 90% of the color in sugarcane industry wastewater using an aged refuse-filled bioreactor. Other technologies, such as thermal electrocoagulation, were used for color removal by Sahu et al. [15] on wastewater from sugarcane industries with a color reduction of 99.7%.

### 2.2. Physicochemical Parameters

Turbidity is a crucial physical parameter of sugarcane industry effluents, as it indicates the concentration of suspended or soluble matter in the water, which can hinder photosynthesis and inhibit aquatic life. Suspended solids are insoluble particles in the effluent, including solid volatile matter that can be biologically degraded, and inorganic matter that requires physical or chemical methods for removal. Soluble matter is the most challenging type of pollution to eliminate, often requiring advanced technologies. Discharging sugarcane industry wastewater into rivers contributes significantly to high water turbidity, which disturbs photosynthetic activity and creates an oxygen imbalance [33].

Industrial effluents from the sugarcane industry also contain mineral pollutants such as magnesium, sodium, sulfur, nitrogen, and phosphorus, which can be present in both dissolved and adsorbed forms. Nitrogen and phosphorus are well-known examples that can lead to eutrophication when discharged into the environment without proper treatment. Prata et al. [34] support that the concentration of nitrogen and phosphorus in sugarcane industry wastewater is low, but it is important to note that even low concentrations of these nutrients can have significant impacts on the environment if they are not appropriately treated. With regard to sulfur, while it is not considered a pollutant at low concentrations, the high levels of sulphate in sugarcane industry effluents can contribute to the production of hydrogen sulfide, which can cause unpleasant odors and have negative impacts on human health and aquatic life [35]. The use of chemical products during the handling and transformation of sugarcane can result in effluents with a high concentration of sodium. This can have negative impacts on soil quality when the effluent is released into the environment. Sodium in the effluent can lead to soil degradation and reduced fertility, as it can cause soil compaction, reduce soil permeability, and increase soil salinity. This can ultimately affect the growth and yield of crops and vegetation in the affected area [36].

The concentration of dissolved gases in sugarcane industry effluents varies with temperature and atmospheric conditions, in addition to dissolved solids. These gases can indicate the occurrence of biological or chemical reactions in the effluent. Dissolved oxygen, which is essential for aquatic life, decreases as a result of the self-purification of effluents.

The organic load of wastewater serves as an indicator of its pollution level, and microorganisms can biologically degrade the organic load by utilizing the carbon in nitrogen molecules for their growth. Various parameters, including BOD_5_ and COD, can be used to characterize the organic load content of sugarcane industry effluents. Sugarcane industry effluents are widely regarded as high-strength wastewater due to their organic load, which can vary significantly depending on seasonal and raw material handling conditions [14,31,37].

### 2.3. The pH and the Temperature

The temperature of wastewater generated from sugarcane industries is not constant and can fluctuate between 20 and 60 °C depending on various factors, such as the stage of the processing and the time of the year. It is important to manage and treat high-temperature wastewater appropriately to mitigate potential environmental and health hazards [14]. The temperature of wastewater after ethanol production can increase up to 80 °C, which has a significant impact on the effectiveness of the treatment process. Microorganisms responsible for organic matter degradation are classified based on their temperature tolerance into three categories: psychrophilic, mesophilic, and thermophilic. It is important to note that before discharging wastewater into the environment, it must be at a specific temperature to prevent harm to aquatic life. In physical or chemical treatment processes, temperature variations can trigger chemical reactions or changes in other physical parameters of the wastewater, such as pH. The pH value is a measure of the acidity or alkalinity of the effluent and can provide information on its aggressive or scaling properties.

The pH levels in distillery effluents tend to be acidic due to the use of chemical products that promote yeast development, whereas the pH of effluents from sugar refineries tends to be over 7, indicating alkalinity. The use of acidic chemicals products in the distillery process can result in acidic wastewater, which must be treated appropriately before being discharged into the environment. On the other hand, sugar production effluents are typically alkaline due to the use of chemicals products that increase the pH during processing. Regardless of the pH level, it is important to monitor and treat all effluents before discharge to prevent negative impacts on the environment and human health [38]. To regulate the pH levels in cane industry effluents, a buffer tank is commonly used. This tank combines wastewater from both the sugar factory and the distillery to neutralize the pH levels. Maintaining a stable pH level is crucial for the growth and development of microorganisms during biological treatment. For cane industry effluents, a pH level of around 7 is typically targeted to ensure stable microbial activity and protect aquatic life.

As self-contained industrial complexes, cane industries typically have integrated maintenance departments. However, these departments often add oil and water from floor and vehicle washing to the effluent, which can make the characterization and disposal of the wastewater even more challenging. These additional contaminants can affect the overall composition of the effluent, making it more complex to treat and dispose of safely. It is important for cane industries to implement proper management strategies to minimize the impact of these contaminants and ensure that the effluent is properly characterized and disposed of to protect the environment and human health [13]. The quantities of oil and fat from floor and vehicle washing added to the effluent in cane industries can be significant and can lead to pump clogging in wastewater treatment plants. It is important to properly manage these contaminants to prevent adverse impacts on treatment processes and the environment. Table 1 provides characteristics of wastewater from sugarcane industries, as reported by several researchers. These characteristics can vary depending on the specific industrial processes used and the location of the industry. Proper characterization and monitoring of the effluent are necessary to ensure that appropriate treatment processes are used to minimize environmental impacts.

**Table 1:** Characteristics of wastewater from sugarcane industries.

Parameters	Sugar Production	Distillery Vinasse + Blanquette	References
Odor	-	Unacceptable	[27,28]
Color (pt-Co)	-	12–17,000	[15,39]
Temperature (°C)	29.3–44.3	46.3–66.3	[14]
pH	6.7–8.4	3.9–4.9	[14,40]
Conductivity (µm/cm)	540.3–925.9	3910–50,500	[41]
BOD_5_ (mg/L)	654.6–1968.5	5000–60,000	[40,42,43]
COD (mg/L)	1100.3–2148.9	16,000–190,000	[12,44,45]
Chloride (mg/L)	30.5–866.6	600.6–7475.7	[46]
Total hardness	356.2–2493.1	3100.3–4477.2	[47,48]
Calcium (mg/L)	365.4–468.0	288.5–3389.8	[40,49]
Magnesium (mg/L)	214.8–341.0	100.3–1828.1	[14,48]
Sodium (mg/L)	-	4–118	[39,42]
Potassium (mg/L)	0.6–2	3000	[45,47]
Total solids (mg/L)	2452.3–3050.6	12876.9–150,300.9	[14,46]
Total dissolved solids (mg/L)	1480.2–1915.1	13,000.0–88,265.1	[50]
Total suspended solids (mg/L)	220.3–790.7	2900.1–150,000.0	[41,50]
Nitrates (mg/L)	0.4–0.9	2.40–32.9	[45]
Organic nitrogen (mg/L)	24.3–36.4	75.2–400.7	[14]
Ammoniacal nitrogen (mg/L)	0.0–4.2	10.9–18.1	[40,45]
Total nitrogen (mg/L)	11.1–40.6	85.8–1355. 3	[14,40]
Phosphate (mg/L)	1.2–9.6	1.2–108	[47,51]
Total phosphorus (mg/L)	1–19	60–250	[47,51]
Sulfate (mg/L)	21.5–51.7	300.0–6050.5	[41,47]
Oils and fats (mg/L)	88.7–134.4	30.3–202.1	[45]

To eliminate this organic and chemical pollution, several treatment methods are used throughout the world, depending on the realities or the technical and financial means.

## 3. Different Technologies for Treating Effluents from Sugarcane Industries

The largest sugar-cane-producing countries, namely, Brazil and India, have been attempting to treat these industrial effluents since the late 1950s, using methods that are not always sustainable. Nevertheless, sugarcane industry effluents, with their elevated nutrient content, can be utilized directly for irrigation. This practice is commonly referred to as fertigation [52]. The recovery of biocompost after the transformation of liquid effluents into organic manure that can be used in agriculture allows for the substitution of chemical fertilizers with biological fertilizers. With a humidity level of approximately 93%, effluents from the sugarcane industry are ideal for biocomposting, as humidity is one of the important operating conditions for biocomposting. However, it should be noted that the high quantity of sugarcane industry effluents makes their biocomposting challenging, with a process that can take up to two weeks. These methods are not without consequences for the environment, as effluents from the sugarcane industry contain pollutants that can degrade the soil [27,53]. However, Christofoletti et al. [52] suggest that soil is not impacted by the use of sugarcane effluents for fertigation when the application rates are below 300 m^3^/ha of vinasse and 4 kg/m^3^ of potassium. Some plants, specifically the fungi family, use vinasse as a source of nutrients for their growth [54]. Another traditional method is evaporation by incineration, which reduces the amount of wastewater, and the byproducts obtained are used as feed for livestock. Its main limitation is its energy consumption, as boilers are used for evaporation and recovery of condensate. Despite their negative impacts on the environment, these traditional methods are still used in many countries due to a lack of regulations.

Besides these so-called traditional methods, several other techniques are utilized for treating effluents from sugarcane industries. Among the most widespread techniques are biological treatments, which do not require any additional chemical products and, thus, occur naturally.

### 3.1. Biological Processes

These biological treatments are a controlled intensification of the phenomena that occur in the soil during fertigation. There are aerobic and anaerobic biological processes. Aerobic processes are suitable for faster and more complete biological degradation [55]. However, their limitations lie in their ability to treat high-strength wastewater, with a chemical oxygen demand (COD) concentration exceeding 1000 mg/L [56]. Aerobic processes require a large amount of oxygen (i.e., energy). That is why most studies on sugarcane industry wastewater treatment have been conducted under anaerobic conditions, in the absence of oxygen, due to their high organic matter load.

Indeed, under anaerobic conditions, the degradation of organic matter generates gases such as CH_4_ and CO_2_, as well as other gases, since the hydrogen acceptor released during degradation can be something other than oxygen. Anaerobic digestion is not fully understood, but the reactions that occur are well known. From hydrolysis to methanogenesis, including acidogenesis and acetogenesis, lipids, proteins, and polysaccharides are transformed into soluble amino acids, sugar, and fatty acids. Several anaerobic biological treatment systems have been identified for the treatment of sugarcane industry effluents.

High-rate reactors, as their name suggests, allow the treatment of large volumes of effluents with high organic loads. It should be noted that these reactors are an evolution of conventional biological treatment systems (biological digestion). The digestion of organic matter is carried out by anaerobic microorganisms in a digester that can be compared to a septic tank or even an Imhoff tank [57]. As the growth rates of these microorganisms are slow, this implies slow reaction times (long hydraulic residence times) or the use of fixed biomass systems, which makes it possible to dissociate the sludge age (growth of the microorganisms) from the hydraulic residence time.

#### 3.1.1. AF (Anaerobic Filters)

This refers to a biological reactor with a fixed-bed biomass for organic matter degradation. The effluent is treated in contact with the filter, which allows for the retention of solid particles. It is mostly used as a post-treatment step due to its technical and economic advantages [58]. The system is composed of two parts. The first part is a sedimentation tank similar to a septic tank, and the second part consists of filters. The second part is divided into several chambers with filtering materials such as ash, specially shaped plastic pieces, gravel, or crushed stone. These materials can be replaced by local materials to reduce costs [59,60]. The shape of the filtering material is very important as the treatment efficiency depends on it. Indeed, the diameter of the filtering material varies from 12 to 55 mm with a surface area of 90 to 300 m^2^ per m^3^ of reactor volume. All of this allows for a long enough contact time between the organic pollution and the filtering bed. The system can be fed by a downward or upward flow. However, in the best-case scenario, the upward flow is recommended to avoid washing out the active biomass. The flow regime is closely related to the hydraulic retention time, which is the most important parameter in the treatment of cane industry effluent by an anaerobic filter system. Bodík et al. [61] achieved a reduction of approximately 66% in COD using an anaerobic filter to treat wastewater. Using the same technology on sugarcane industry effluents, Fito et al. [51] achieved a 65% COD removal at a concentration of 10 g/L and an HRT of 10 days. Cabrera-Díaz et al. [62], in treating sugarcane industry effluents, showed in their study that anaerobic filters can achieve a COD removal of around 75% at an HRT of 5 days and a methane production of about 0.315 L CH_4_/g COD.

#### 3.1.2. UASB (Upflow Anaerobic Sludge Blanket)

This is one of the most commonly used reactors for the treatment of sugar industry wastewater. The activated sludge is in granular form, which has excellent settling properties. The influent enters the reactor in an upward and vertical flow, thus playing the role of recirculation [63]. All of this gives the UASB reactor an advantage in terms of investment cost compared to other reactors. However, its limitation lies in its relatively long acclimatization period, requiring a substantial amount of sludge and a long HRT. Chan et al., Fuess et al., and Mohana et al. [46,57,64] suggested that in the case of wastewater highly loaded with organic matter such as sugarcane industry effluents, the UASB reactor is not suitable as it does not allow efficient degradation of organic matter. Lettinga et al. [65] demonstrated that industrial wastewater with a high organic load up to 25 g DCO/L/d can be effectively treated by UASB with promising removal rates. At thermophilic temperatures ranging from 45 to 65 °C, an average removal of about 50% was obtained by Contreras–Contreras et al. [66] using a UASB to treat wastewater from sugarcane industries. The wastewater from sugarcane industries is highly loaded, and for organic loads above 20 gCOD/L/d, the COD removal efficiency remains below 75% with treatment using UASB [67,68,69].

#### 3.1.3. EGSB (Expanded Granular Sludge Bed)

The EGSB is a reactor similar to the UASB but with an extended version capable of handling higher loads. The main difference between the EGSB and the UASB lies in its ability to separate biomass, effluent, and biogas produced with better contact between granular sludge and wastewater [70]. The production of carboxylate can be improved during dark fermentation of vinasse in an EGSB by varying certain parameters such as HRT or substrate concentration [71]. During anaerobic treatment with an EGSB, Vaquerizo et al. [44] achieved 76% reduction in COD at a temperature of 26 °C and a methane production of 4.2 CH_4_/L/d with a COD concentration of 7 g/L. At an organic loading rate of 12.6 gDCO/L/d, Mohana et al. [46] treated effluents from sugarcane industries with an EGSB, achieving a removal efficiency of around 80% at a HRT of 2 days and a temperature of 20 °C. Other similar studies reported removal rates of 68% and 76%, respectively, by Cruz-Salomón et al. and López et al. [72,73].

#### 3.1.4. ASBR (Anaerobic Sequencing Batch Reactor)

This technology is new compared to conventional biological treatments mentioned above, with the advantage of being flexible for various effluents. It is a batch system, meaning that feeding is performed at highly variable organic loads, thereby facilitating good degradation of organic matter. The effective settling of organic matter is promoted by a low organic loading at the end of the cycle [74]. ASBR operates cyclically with a sequence of feeding, reaction, settling, and discharge, maintained by a high feeding rate at the beginning of the cycle. This system also allows for the recovery of biogas [75]. After feeding, a gentle agitation is performed to ensure contact between the effluent and the purifying sludge. This gentle agitation allows for settling and the production of biogas resulting from the degradation of the effluent by the bacteria contained in the purifying sludge. It is a reactor that allows for the separation of HRT and SRT [76]. This operation for sugarcane industry wastewater treatment is not very developed [77,78]. However, Sara et al. [79] used this technology to treat wastewater from sugarcane industries, with interesting COD removal efficiencies ranging from 81 to 91% with an inlet load of 0.25 gCOD/L/d. By adding a recirculation loop to the ASBR, the COD and ammonium removal efficiencies can be higher than the conventional ASBR system. When treating wastewater from the sugarcane industry, Kee et al. [80] obtained removal efficiencies of 52% and 31% for COD and ammonium, respectively, using the modified ASBR, compared to 31% and 17% for the conventional ASBR.

#### 3.1.5. AFBR (Anaerobic Fluidized Bed Reactor)

The organic pollution is treated using a fixed-bed system. The purifying biomass is fixed onto small-diameter material such as activated carbon or fine sand. In this reactor, the flow can be either upward or downward. For better treatment and bacterial growth, the upflow configuration is recommended for organic pollution removal [46,81]. The upward flow maintains the fluidization of the bacterial bed and effluent thanks to the drag forces, preventing biomass washout. In addition to low clogging, one of the major advantages of treating sugarcane industry wastewater with a fixed bed anaerobic reactor system is the maintenance of the purifying biomass on the fixed bed thanks to the flow of the upward stream [82]. This is a system that allows for the degradation of organic matter and the recovery of biogas, either methane or hydrogen [83,84]. At a temperature of 30 °C and an HRT of 24 h, Siqueira et al. [85] achieved a removal of approximately 51% of COD by applying an anaerobic fluidized bed reactor to effluents from sugarcane industries. In a sugarcane industry wastewater treatment system with an anaerobic fluidized bed reactor, decreasing the HRT will result in better methane production rates of up to 0.3 LCH_4_/gCDO/L/d. Similarly, at thermophilic temperatures (55 °C), the elimination rate of DCO can reach up to 71% at an organic load of about 24 gDCO/L/d [84].

Activated sludge and macrophyte lagooning are also used for the purification of sugarcane industry wastewater, with interesting pollution removal rates. The COD and BOD_5_ can be removed at more than 90% with an activated sludge system when treating sugarcane industry wastewater [86]. Studies conducted by Fonkou et al. [87] on the treatment of distillery wastewater using Echinochloa pyramidalis planted filters yielded good results. Indeed, the following removal rates were achieved: 80% for conductivity, 90% for color, 79% for suspended solids, 60% for COD, 90% for BOD_5_, 79% for total nitrogen content, and 50% for total phosphorus content.

The discharge of wastewater from sugarcane industries after biological treatment often does not meet the discharge standards, representing a potential danger to the environment. Therefore, there is a need for the implementation of an efficient treatment process. Physicochemical treatment processes appear suitable for the elimination of mainly mineral pollution in sugarcane industry wastewater. However, their limitations are the high costs of treatment and the additional costs of treating the byproducts [88].

Table 2 gives the operational parameters and treatment performance for sugarcane wastewater treatment.

**Table 2:** Operational parameters and treatment performance for sugarcane wastewater treatment.

	Scale	T	Operating Conditions	COD Removal	Methane Yield	COD Effluent	Duration	Ref.
	(L)	(°C)		(%)	(m^3^-CH_4_·g^−1^ COD_removed_)	(mg·L^−1^)	(d)	
Two-stage UASB	7–14	23.5–55	pH 7.0	15–44	0.23	29,817	475	[89]
			HRT 48–17					
			OLR 5.5–22					
One-stage AnSTBR	1.65	55	pH 3.8	6.4	-	7308	392	[90]
			HRT 4					
			OLR 60					
One-stage UASB	15	26–52	pH 6.6–8.3	76	0.11	41,700	72	[91]
			HRT 96					
			OLR 0.2–1.7					
Two-stage CSTR	5	38	pH 7.29	-	0.22	4030–5670	90	[92]
			HRT 924–600					
			OLR 2.1–3.2					
One-stage UASB	3.5	35	pH 7–7.5	90	0.268	65,180	75	[93]
			OLR 7.6–12					
			HRT 116.64					
			ULV 0.1					
One-stage UASB	40–21	20–30	pH 6.5–6.8	49–82	0.181–185	45,000	230	[94]
			OLR 0.2–7.5					
			HRT 67.2–43.2					
			ULV 0.019–0.018					
One-stage UASB	13.7–10.6	55	pH 4.6–8.6	90	0.274	45,000	387	[95]
			OLR 0.15 to 3.50					
			HRT 38.4–67.2					
			SRT 23–267					
Two-stage UASB	214.2–115	45	pH 6.5–7.5	40	-	12,800–45,000	100	[96]
			OLR 6					
			HRT 24–12					
Two-stage AFBR	0.743	30–55	pH 3.9–4.6	-	-	5000	240	[97]
			OLR 15–120					
			HRT 8–1					
One-stage APBR	2.3–3.5	55	pH 4.6–6.5	26.2–33.3	-	35,000	30	[98]
			OLR 36.2–54.3					
			HRT 24–8					
One-stage UASB	6	30	pH 4.5–7.22	69	0.263	121000	70	[99]
			OLR 17.05					
			HRT 7.5					
One-stage AnSBBR	3.5	35–55	pH 6.9–8	79–82	0.304–0.352	1000–5000	175	[100]
			OLR 1.5–7					
			HRT 8					
Anaerobic Hybrid reactor	-	50	pH 7	79	0.52	90,000–130,000	-	[101]
			OLR 8.7					
			HRT 120					
One-stage AFBR	4.192	30	pH 6.5–8.3	51–70	0.212	2273–20073	355	[85]
			OLR 3.33–26.19					
			HRT 24					
			ULV 76					
One-stage APBR	2.3	25	pH 4.6–6.5	37–40	-	35000	30	[102]
			OLR 36.2					
			HRT 24					
One-stage UASB	1.5	40	pH 4–9	79	0.239	22,000–23,000	200	[103]
			OLR 6.1–9,6					
			HRT 86.4–60					
One-stage UAF	3.4	29	pH 7.5	75.1	0.315	50,000	180	[62]
			OLR 10					
			HRT 120					
One-stage APBR	87.5	35	OLR 4.4	86.7	0.207	-	-	[104]
			HRT 48					
One-stage AFBR	300	30–37	pH 5.05–7.35	60–70	0.386	60,000–70,000	-	[105]
			OLR 20					
			HRT 48–103.2					
One-stage AFBR	-	35	OLR 24.32	88	7.72	-	-	[106]
			HRT 16.8					
One-stage FBR	10,000	37	pH 7	60–73	0.288	51,000–57,000	220	[107]
			OLR 9.2					
			HRT 79.2–60					
One-stage EGSB	3.3	26	pH 4.5–7	76	0.244	71,605	60	[72]
			OLR 5.4					
			HRT 168					
One-stage EGSB	12	31	pH 4.04–5.35	68	2.57	40,000–80,000	180	[73]
			OLR 5.7					
			HRT 206.4					
Two-stage FBR + CSTR	-	35	pH 7.5–8.2	67	0.315	45,000–60,000	365	[108]
			OLR 21.3					
			HRT 96					
Two-stage CSTR + FBR	0.8	37	pH 7.5	92	0.33	61,000	70	[109]
			OLR 4.1					
			HRT 120					
Two-stage APBR + ASTBR	2.3	55	pH 5.5–7.5	89	0.319	28,300	240	[64]
			OLR 25					
			HRT 180					
Two-stage UASB	5.6–12.1	54–56	pH 6.59–7.7	60	0.2	44,500	160	[67]
			OLR 45					
			HRT 24					
One-stage UASB	120	22	pH 4.2–7.75	90	0.299	19,220	700	[110]
			OLR 20					
			HRT 792					
One-stage EGSB	-	20	OLR 12.6	80	-	110,000–190,000	-	[46]
			HRT 48					

OLR = organic loading rate (kg·m^−3^·d^−1^); ULV = upflow liquid velocity (m·h^−1^); HRT = hydraulic retention time (h); SRT = sludge retention time (d).

### 3.2. Physicochemical Treatment Systems

Sugarcane industries wastewaters are loaded with various particles due to cane cutting, resulting in a high volume of suspended solids in the effluent. Coagulation–flocculation can then be used to remove suspended matter.

#### 3.2.1. Coagulation–Flocculation

This is one of the most common physicochemical treatment systems. Suspended solids and colloidal matter are gathered into flocs using a coagulant. Parameters such as pH, temperature, time, and/or agitation speed can influence coagulation. The most commonly used coagulants in the treatment of sugarcane industry effluents are chemical polymers, aluminum salts, or natural coagulants. According to Freitas et al., Matilainen et al., and Sher et al. [111,112,113], the most commonly used coagulant salts are aluminum sulfate (Al_2_(SO_4_)_3_), ferric sulfate (III) (Fe_2_(SO_4_)_3_), AlCl_3_, and ferric chloride (FeCl_3_). Activated carbon has a very high specific surface area, which makes it an advantageous choice as a coagulant. Pollutants are trapped in the pores of activated carbon, which are formed by heating the material at very high temperatures in the absence of oxygen [114,115]. Studies by Garika et al. [48] have concluded the comparative effectiveness of natural coagulants compared to chemical coagulants at a concentration of 0.25 g/L with a reduction of about 99.2% in color for the treatment of sugar industry effluents. The use of sugarcane bagasse as a coagulant for wastewater treatment is growing with very promising results [116,117].

#### 3.2.2. Advanced Oxidation Processes (AOPs)

The degradation of pollution is achieved by species generated through chemical reactions, which are called OH° radicals. These species are produced during the first oxidation step of ozone, hydrogen peroxide, or ultraviolet radiation. The second step involves the reaction of the free radicals with the pollution in order to eliminate it [118,119]. In the homogeneous phase, we have H_2_O_2_/Fe^2+^ and H_2_O_2_/O_3_. In the heterogeneous and/or homogeneous phase, they are called photocatalytic, and we have H_2_O_2_/UV, O_3_/UV and Fe^2+/^H_2_O_2_/UV, TiO_2_/UV. Two other advanced oxidation processes are electrochemical and sonochemical [120].

Apollo et al. [121] achieved a degradation of color and COD of 88% and 85%, respectively, using UV photodegradation to treat distillery effluents with an initial COD concentration of 5 g/L. Electro-Fenton, on the other hand, is more effective in removing color with values up to 90%, but these values are moderate for COD removal [122]. The main limitations of advanced oxidation processes are the presence of radical inhibitors. These are compounds that react with the OH° radical without producing the superoxide radical. Another chemical process for the treatment of sugarcane industry effluents is ion exchange. It is similar to ion chromatography in that it allows ions to be fixed on a stationary medium based on their charges [123]. One advantage of this method over others is its ability to degrade color and inorganic pollution. However, at high loadings, its efficiency is reduced [124]. Pattakamol et al. [125] obtained interesting removal rates of organic matter by using an amberlite ion exchange and magnetic resin in the treatment of sugarcane industry effluents. Adsorption is indicated as a treatment process for sugarcane industry effluents due to the advantages it offers. It should be noted that it is less expensive than most physicochemical processes, because the materials used for adsorption are very accessible. Furthermore, these absorbent materials and byproducts have no harmful effects once they are discharged into the environment [126]. Using activated carbon as an adsorbent can reduce pollution in sugarcane industry wastewater by up to 40% [51].

Membrane processes such as reverse osmosis, nanofiltration, ultrafiltration, and microfiltration are also used to treat effluents from sugarcane industries. These processes involve the use of membranes with different pore sizes to separate pollutants from the wastewater. They are effective in removing dissolved solids, suspended solids, and other contaminants from the effluent, producing a high-quality treated water. However, these processes can be energy-intensive and require regular cleaning and maintenance to prevent fouling and scaling of the membranes.

#### 3.2.3. Membrane Processes

The treated effluent passes through a barrier. Depending on the applied force, the characteristics of the barrier, and the components of the water, such as chemical pollutants, solid matter, and colloids, molecules are retained. One such system is nanofiltration, which is capable of retaining multivalent ionized salts such as sodium, calcium, sulfates, magnesium, and nonionized organic compounds with a molar mass greater than 250 g/mol. Three principles are involved in this separation: electrostatic rejection, solubility–diffusion, and screening through micropores [127]. At a molecular weight cutoff of 200 to 1000 Daltons, nanofiltration membranes can have pore sizes ranging from 0.5 to 10 nm under a pressure of up to 30 bars, depending on the treatment [127,128]. Nanofiltration is a technology situated between reverse osmosis and ultrafiltration. Reverse osmosis is one of the most well-known membrane processes due to its use for desalination of seawater. With a pore size less than 0.5 nm, a significant membrane pressure of up to 60 bars is required to retain solutes such as salts and amino acids with a molecular weight cutoff below 1000 Daltons [129,130]. The microfiltration and ultrafiltration are also membrane processes used in the treatment of effluents from sugarcane industries. The principle highlighted is essentially sieving separation, with pore sizes ranging from 0.1 to 5 nm for microfiltration and from 1 to 100 nm for ultrafiltration. Separation of solids and colloids up to proteins and large molecules for ultrafiltration is ensured at a transmembrane pressure between 1 to 10 bars.

These are expensive and energy-intensive technologies, which makes their applications challenging. Nevertheless, studies have been carried out on sugarcane effluents. A C–MF–NF (coagulation microfiltration and nanofiltration) combination was used to treat vinasse by Lebron et al. [42] with removal rates of COD, color, and ions of around 90%. By replacing the microfiltration membrane with a nanofiltration membrane, Silva et al. [43] used the same integrated system (UF–NF), but with and without precoagulation to treat vinasse. The results concluded that the use of a coagulant in pretreatment influences the removal of COD and color. Other membrane technologies, which are still very interesting, exist, but their application to sugarcane industry effluents is not widespread. These include electrodialysis, distillation, membrane evaporation, and pervaporation.

Membranes used for separation in the treatment of effluents from sugarcane industries by membrane processes can be classified into two categories: organic and inorganic membranes. Organic membranes are manufactured using polymers such as polypropylene (PP), polyvinylidene fluoride (PVDF), polyethersulfone (PES), polysulfone (PS), polyamide (PA), or cellulose acetate (CA). It should be noted that inorganic membranes offer more advantages in terms of chemical resistance and temperature compared to organic membranes; however, the latter category remains more accessible due to their costs [131].

The natural, biological treatment systems alone are not sufficient to guarantee zero or positive impact on the environment. With regard to physicochemical treatment systems, the additional costs of treating the generated byproducts limit their use. Membrane filtration, on the other hand, allows for very high-quality effluent, but the water to be treated must have certain characteristics. Since the effluents from the sugarcane industry are wastewater heavily loaded with various pollutants and have a high risk of rapid membrane fouling, it is therefore necessary to seek treatment systems that are effective in reducing color, organic matter, and especially mineral pollutants.

## 4. Perspectives

The membrane bioreactor is a hybrid system that integrates biological and physical treatment processes. It combines a biological reactor, responsible for the degradation of organic matter, with a membrane filter that effectively retains both inorganic and organic matter, as well as highly resistant pollutants. It exhibits remarkable selectivity as it effectively retains pollutants regardless of their degree of flocculation. The initial applications of MBR technology were conducted in the United States by Rensselaer Polytechnic Institute of Troy and Dorr Oliver Inc. of Milford, Connecticut. Although the technology was developed in the 1960s, it was not until 1990 that it started being widely employed for industrial wastewater treatment purposes [132,133]. Since then, numerous studies have been conducted on various types of effluents, consistently yielding interesting results. The growing adoption of membrane bioreactors for industrial effluent treatment can be attributed to the multiple advantages they offer over other treatment systems. One of these advantages is the MBR’s ability to simultaneously treat water through the biological reactor and clarify it through the membrane section. This dual functionality is particularly beneficial for the treatment of sugarcane industry effluents, which often exhibit highly variable organic loads. The MBR enables continuous treatment without compromising the purification performance of the system. Additionally, MBRs help address major challenges associated with conventional treatment systems, such as sludge production and space requirements, by reducing them significantly [134,135,136].

In addition to valorization in agriculture, wastewater treated by a membrane bioreactor can be used in the manufacturing circuit, all thanks to the high selectivity of the membrane barrier. Furthermore, it is important to highlight that the hydraulic retention time (HRT) and sludge retention time (SRT), which are often misconstrued, are distinct and well separated in MBR treatment. This separation facilitates the optimal development of biomass within the system [137].

The hydraulic retention time can be significantly reduced in a membrane bioreactor (MBR), enabling the treatment of large volumes of effluent within a shorter timeframe compared to conventional biological treatment. However, operating a membrane bioreactor does come with certain limitations. One such limitation is the relatively high operating costs, primarily due to the need for regular monitoring and maintenance of the system.

Another significant challenge faced by MBRs is membrane fouling. Despite efforts to control fouling, it remains a prominent issue during MBR operation. Fouling occurs when particles, organic matter, and other substances accumulate on the membrane surface, compromising its permeability and overall efficiency. Addressing membrane fouling is an ongoing concern in MBR systems, requiring strategies such as chemical cleaning, backwashing, membrane replacement, or the use of advanced membrane materials to mitigate its impact and maintain optimal performance [132,138].

Operating costs are even higher when the bioreactor is in an external loop. To overcome the high operating costs when starting up a membrane bioreactor, some researchers recommend treating the effluent under anaerobic conditions. An anaerobic membrane bioreactor allows for the recovery of biogas that can be valorized as energy, thus allowing for return-on-investment calculations. In anaerobic conditions, the membrane bioreactor can support relatively high organic loads compared to aerobic membrane bioreactors. Since the effluents from cane industries are heavily loaded with organic matter, an anaerobic membrane bioreactor would allow for the recovery of significant quantities of biogas, which can be valorized as energy [139]. At an organic load of COD ranging from 2.5 to 6 kgCOD/m^3^/d, a biogas production of around 6.4 Nm^3^ of CH_4_/m^3^ was obtained by Santos et al. [49] during treatment of sugarcane industry wastewater by a membrane bioreactor.

It should be noted that several computational simulation models are used to forecast the treatment process in membrane bioreactors. Computational simulations play a significant role in reducing costs and optimizing treatment operation time [140,141].

Table 3 gives the performance of the membrane bioreactors applied to sugarcane and agro-food industry effluents.

**Table 3:** Performance of membrane bioreactors applied to sugarcane and agro-food industry effluents.

Reactor	Wastewater	COD (mg/L)	T °C	HRT	Removal COD (%)	LCH_4_/gCOD	References
AnBRM	Sugarcane	13,147	22	2.58	97.5	-	[142]
AnBRM	Sugarcane	46,000	37	14	92	0.37	[143]
BRM	Sugarcane	35,200	4	7	40	-	[144]
BRM	Sugarcane	17,677	22	3.6	85	-	[47]
AnBRM	Sugarcane	16,706	25	3.1	97	>0.32	[49]
AnBRM	Synthetics	4000	35	2	92	0.6	[145]
AnBRM	Food waste	26,000	22	13	86	0.3	[146]
AnBRM	Dairy industry	102,346	55	2.5	98.8	0.31	[147]
AnBRM	Swine wastewater	7400	37	27	86	0.4	[148]
AnBRM	Agro-industry	20,900	36	25	98	0.15	[149]
AnBRM	Fast food	17,500	40	4.3	97	0.4	[150]
BRM	Brewery	1710	-	-	87	-	[151]
AnBRM	Brewery	15,500	35	3.5	99	0.11	[152]
AnBRM	Food waste	73,670	-	30	99	0.51	[153]

Indeed, depending on whether it is anaerobic or aerobic, the bioreactor can be configured in two different ways.

## 5. Configuration of Membrane Bioreactors

Since its development, the membrane bioreactor has been of the external loop configuration, meaning that the membrane is installed outside the biological reactor in a cartridge (membrane module) [154]. Membrane fouling is controlled by a high-speed circulation of the biomass or by chemical washing if necessary.

**Figure 2:**
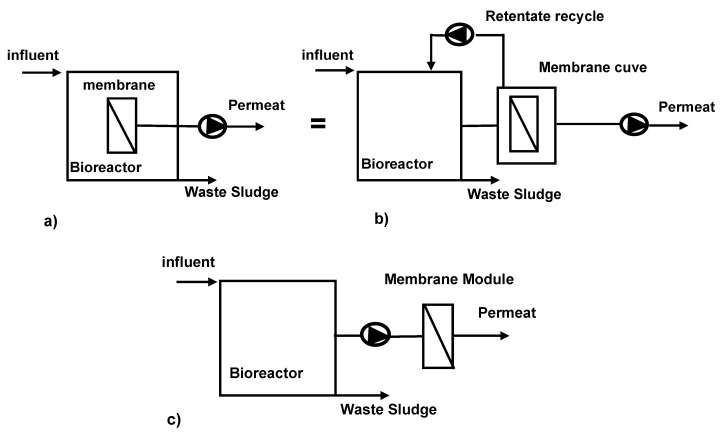
Membrane bioreactor configuration (**a**); (**b**) immersed MBR; (**c**) external MBR.

One significant advantage of external loop MBRs is their capability to treat high-strength effluents at velocities ranging from 0.5 to 4 m/s, even under high temperatures and pH conditions [155]. Indeed, some researchers report that the external loop configuration of the membrane bioreactor is not suitable for treating effluents with high organic loads. The main reason is that the external membrane is more susceptible to fouling, which can lead to a decrease in the efficiency of the treatment process. Furthermore, the external configuration requires a higher hydraulic pressure to circulate the effluent, which can increase the energy consumption of the process. Based on these considerations, certain researchers propose that the immersed internal configuration of the membrane bioreactor, wherein the membrane is positioned inside the bioreactor tank, is better suited for treating high organic loads. This configuration results in lower hydraulic pressure and reduced fouling risk, ultimately leading to a more efficient and cost-effective treatment process [156]. At high organic loads, the shear rates and operating costs are quite high [157]. To ensure adequate filtration flow, tangential filtration is indicated for this configuration, and the membranes used are either flat or tubular. In order to minimize operating costs, an alternative configuration known as the immersed membrane bioreactor has been developed. In this setup, the membrane is submerged within the treated biomass using flat or hollow fiber modules [155]. Indeed, compared to the external loop BRM, the immersed BRM has been commercially successful. This is mainly due to its lower operating costs and simpler design. The immersed configuration involves submerging the membrane directly into the mixed liquor, eliminating the necessity for a separate tank for the membrane module. This configuration offers the advantage of lower energy consumption as it requires reduced pumping requirements. However, immersed BRMs may face some operational challenges, such as membrane fouling, which can lead to a decrease in permeability and require more frequent cleaning. Gander et al. and Shin et al. [137,158] suggest that fouling is better controlled in an immersed MBR configuration, which is suitable for treating low-loaded effluents. Consequently, frontal filtration can be implemented with relatively low permeate fluxes. Membranes can be categorized as either organic or inorganic. Organic membranes are more cost-effective but have lower resistance compared to inorganic membranes. Ceramic membranes are the most frequently employed inorganic membranes, while metal membranes are gaining popularity due to their superior resistance to fouling and ability to withstand high temperatures [159,160]. The immersed external membrane bioreactor (EMBR) aims to leverage the advantages of both external and immersed membrane configurations. By locating the membrane outside the reactor in a separate tank, the EMBR enhances shear stress on the membrane surface, leading to improved permeate flux. Furthermore, this configuration allows for convenient chemical cleaning of the membrane. The EMBR is capable of handling high-strength wastewaters as well as accommodating variable wastewater flows and compositions, thereby making it a versatile and efficient treatment option [161]. A schematic representation of the membrane bioreactor is given in Figure 2. While all these parameters are crucial in the design of the membrane bioreactor, it is essential to emphasize that its operational efficiency is closely tied to the interconnected biological and physical conditions it necessitates.

## 6. Operating Conditions

Some of the operating conditions of a membrane bioreactor include permeate flux, pH, temperature, and fouling. pH is a critical parameter as it directly impacts the activity of microorganisms within the reactor. Membrane bioreactors are designed to operate at a neutral pH. However, industrial effluents often have extreme pH levels, and the wastewater from the sugarcane industry is no exception. To address this, various chemicals are employed to neutralize the pH within membrane bioreactors. In a study on effluents from sugarcane industries, Fito et al. [40] used HCl and NaOH to neutralize the pH in order to promote the degradation of pollutants. At thermophilic temperature in a UASB, Harada et al. [68] used a solution of NaHCO_3_ at 5 g/L to neutralize the pH in effluents from sugarcane industries. Lime is commonly used in other sugarcane industries to neutralize the pH.

Temperature directly impacts the biological activity within a membrane bioreactor. Lower temperatures result in reduced purification efficiency, leading to a decrease in COD removal [162]. Indeed, the temperature in membrane bioreactors differs depending on their configuration. An ambient temperature around 20 to 30 °C is suitable for aerobic membrane bioreactors. On the other hand, the temperature must be around 30 °C in an anaerobic membrane bioreactor to promote biological activity and biogas production [134]. Temperature has a significant impact on the treatment of vinasse for the removal of polyphenols in an anaerobic reactor [66]. In the mesophilic temperature range, an anaerobic membrane bioreactor treating sugarcane industry wastewater can achieve organic matter removal rates exceeding 90% [163].

Membrane fouling is considered one of the crucial operating conditions in a membrane bioreactor [164]. Fouling involves two phenomena: adsorption and polarization. In case of physicochemical interaction between the membrane surface and the solutes, a chemical cleaning is mandatory, which is referred to as fouling by adsorption. Fouling by polarization, on the other hand, involves the formation of a second layer, resulting in a diffusion flux of the membrane towards the solute [165]. Backwashing is sufficient to remove the fouling layer. Fouling is closely related to transmembrane pressure and filtration flux. During filtration through a porous membrane, a transmembrane pressure is applied, which can be fixed and may vary depending on the pressure gradient or the permeate flux [166]. The accumulation of soluble compounds, macromolecules, or microorganisms on the surface of the membrane can lead to a horizontal increase in PTM without a significant reduction in filtration flux [167]. During the treatment of effluents from the sugarcane industry, the transmembrane pressure can reach up to 4 bars due to high organic and inorganic loads. This membrane pressure is given by the following equation with Pa (feed pressure), Pc (concentrate pressure), and Pp (permeate pressure):

$$PTM=\frac{Pa+Pc}{2}-Pp$$

Indeed, fouling and TMP are closely related to the permeate flux, which is simply the flow rate of the effluent to be treated relative to the filtering membrane surface area:

$$JW=\frac{Qp}{A}$$

However, membrane fouling becomes significant at a PTM of 0.5 bar with a microfiltration membrane for vinasse treatment using an anaerobic membrane bioreactor. It is necessary to clean the membrane, which can be performed physically or chemically [47]. Indeed, the higher the organic load, the more easily the membrane becomes clogged [168]

In addition to all these operating conditions, the hydraulic retention time (HRT) and solids retention time (SRT) are governing factors that determine the duration of the treatment process. They are the biological parameters in the operation of a membrane bioreactor. The hydraulic retention time is the time it takes for a water droplet to move from its entry into the membrane bioreactor to its exit. Seyhi et al. [169] defined the HRT as the time that microorganisms have to degrade pollutants. The hydraulic retention time depends on the membrane configuration and the organic load of the effluent. It is relatively low in an aerobic membrane bioreactor. In an anaerobic configuration, the HRT is longer and can be up to 30 days [134,170,171]. The HRT is always set according to the desired treatment efficiency. It is the ratio of the reactor volume to the effluent volume:

$$HRT=\frac{Vreacteur}{Qeffluent}$$

Indeed, the HRT is also closely related to fouling. Treatment of vinasse in an anaerobic membrane bioreactor with a reduced HRT leads to an increase in volatile suspended solids, as well as extracellular polymeric substances (EPS) and the average mass of soluble microbial products (SMP). All of this contributes to increased fouling [49]. In the MBR, pollutant degradation is carried out by a purifying biomass. The biomass can be characterized and quantified to ensure the absence of oxygen deficit. Periodic sludge purging is necessary to maintain a proper biomass concentration. The ratio of the reactor volume to the volume of purged sludge corresponds to the SRT. The process of sludge purging enables the renewal of the purifying biomass. This is known as sludge age as it corresponds to the time required for the degradation of certain particulate compounds. The SRT is dependent on the concentration of the purifying biomass [172,173]. By treating brewery wastewater using a membrane bioreactor at a TSS concentration of 9 g/L and an SRT of 60 days, Sawadogo et al. [164] achieved COD removal rates greater than 90%. In a membrane bioreactor using microalgae as the purifying biomass, a decrease in HRT leads to an increase in biomass [174].

$$SRT=\frac{Vreacteur}{QSludge\;discharge}$$

The selection of these operating conditions is crucial for monitoring the treatment of effluents from the sugarcane industry in a membrane bioreactor, regardless of its configuration.

Despite all the advantages offered by membrane bioreactors, there are still some limitations, including membrane fouling caused by the accumulation of solid materials, mineral deposits, or biofilm, leading to reduced treatment efficiency and requiring frequent membrane cleaning or replacement [160,175]. Lastly, these systems require a continuous energy supply for pump and aeration operation, resulting in significant energy consumption [176,177].

## 7. Conclusions

The choice of treatment methods for sugarcane industry wastewater has traditionally been guided by the effluent quality, as well as the presence or absence of pretreatment or secondary treatment systems. While most biological processes have been found to be effective for treating sugarcane industry wastewater, their ability to remove inorganic pollutants is limited. The use of physicochemical processes for treating sugarcane industry wastewater has been hindered by the requirement for a secondary or tertiary treatment system, depending on the selected process. This review of the literature highlights the various processes used for treating sugarcane industry wastewater along with their respective limitations. Due to the high organic content of sugarcane industry wastewater, previous studies have focused on the use of anaerobic biological treatments, which promote methanogenesis. To achieve a circular economy and promote a water, energy, and food nexus, a membrane bioreactor could be an ideal treatment option for sugarcane industry wastewater. The treated wastewater can be used for sugarcane irrigation or even recycled back into the industrial process with energy recovery. Future research should prioritize the assessment of operational conditions impact on the performance of membrane bioreactors in treating wastewater from the sugarcane industry.

## Data Availability

Not applicable.
